# Correlates of individual variation in the porphyrin-based fluorescence of red-necked nightjars (*Caprimulgus ruficollis*)

**DOI:** 10.1038/s41598-019-55522-y

**Published:** 2019-12-13

**Authors:** Carlos Camacho, Juan José Negro, Iraida Redondo, Sebastián Palacios, Pedro Sáez-Gómez

**Affiliations:** 1 Department of Evolutionary Ecology, Estación Biológica de Doñana (EBD-CSIC), Av. Américo Vespucio 26, 41092 Seville, Spain; 2 Department of Biology, Centre for Animal Movement Research (CAnMove). Lund University. Ecology Building, 223 62 Lund, Sweden; 3 Department of Evolutionary Ecology, Museo Nacional de Ciencias Naturales (MNCN-CSIC), José Gutiérrez Abascal 2, 28006 Madrid, Spain; 4 Monitoring Team of Natural Processes (ICTS-RBD). Estación Biológica de Doñana (EBD-CSIC), Av. Américo Vespucio 26, 41092 Seville, Spain; 5 Department of Integrative Sciences, University of Huelva, Campus Universitario El Carmen, Av. Andalucía, 21071 Huelva, Spain; 6 Instituto Multidisciplinar para el Estudio del Medio “Ramón Margalef”, Universidad de Alicante, 03080 Alicante, Spain

**Keywords:** Sexual selection, Behavioural ecology

## Abstract

Many nocturnal animals, including invertebrates such as scorpions and a variety of vertebrate species, including toadlets, flying squirrels, owls, and nightjars, emit bright fluorescence under ultraviolet light. However, the ecological significance of this unique coloration so attached to nocturnality remains obscure. Here, we used an intensively studied population of migratory red-necked nightjars (*Caprimulgus ruficollis*) to investigate inter-individual variation in porphyrin-based pink fluorescence according to sex, age, body condition, time of the year, and the extent of white plumage patches known to be involved in sexual communication. Males and females exhibited a similar extent of pink fluorescence on the under-side of the wings in both juvenile and adult birds, but males had larger white patches than females. Body condition predicted the extent of pink fluorescence in juvenile birds, but not in adults. On average, the extent of pink fluorescence in juveniles increased by ca. 20% for every 10-g increase in body mass. For both age classes, there was a slight seasonal increase (1–4% per week) in the amount of fluorescence. Our results suggest that the porphyrin-based coloration of nightjars might signal individual quality, at least in their first potential breeding season, although the ability of these and other nocturnal birds to perceive fluorescence remains to be unequivocally proven.

## Introduction

Birds are visually oriented animals. In contrast to other vertebrate classes where numerous blind representatives exist, there are no blind species among birds. Birds are known for their excellent ability to discriminate visual detail and, with only a couple of exceptions^1,2^, use the visual channel to acquire information from the environment, guide their movement, and communicate with others. Many bird species are tetrachromats, and see beyond the visual sensitivity spectrum of humans (400–700 nm) through photoreceptors that allow some species to see in a portion of the ultraviolet (UV) range (300–400 nm)^3^. Accordingly, the color palette and the color-producing mechanisms in birds are numerous: they commonly use melanins and carotenoids, but also produce colors and patterns by structural phenomena, such as iridescence^4^, and may even apply cosmetics to themselves^5,6^. Birds are also known to deposit porphyrins in their plumage^7^, a group of pigments produced as an intermediate product during the synthesis of heme^8,9^. However, porphyrin-based colorations are comparatively rare.

Most bird species known to have porphyrins are nocturnal or crepuscular, although there are notable exceptions. Since the early works by Derrier and Turchini^10^, and Völker^11^, it is known that owls (Strigiformes, 239 species), bustards and allies (Otididae, 26 species), and nightjars, potoos and frogmouths (Caprimulgiformes, 119 species) emit pink fluorescence when their feathers are illuminated with a UV light, and that the pigments responsible for this type of fluorescence are porphyrins. More specifically, coproporphyrin III has been identified as the specific type of porphyrin present in the feathers of all bird species for which chemical analyses have been carried out^12,13^. Fluorescence under UV light is known in a wide array of nocturnal animals, including the evolutionary ancient scorpions^14^, some toadlets^15^, and flying squirrels^16^, but in these cases, color producing mechanisms other than porphyrins seem to be involved. The ecological significance of porphyrin-based coloration remains obscure^17^.

The function of porphyrins in the plumage of birds also remains largely unknown and understudied. These pigments usually co-occur with melanin pigments in the same feather, as in owls, nightjars and allies, as well as in young *Elanus* kites^18^, or just give a salmon-pink color to down feathers in bustards and allies^12^. Porphyrins are photo-labile, and the visible color of bustards disappears in just minutes when exposed to sunlight^12^. The ephemeral nature of the porphyrins may explain why they are mainly found in nocturnal animals, but it does not readily inform what their function(s) may be^17^.

For instance, porphyrins in the juvenal plumage of the crepuscular *Elanus* species are considered a form of camouflage that disappears quickly as soon as young fledge^18^. For the porphyrins found in the internal feathers of bustards, Galván *et al*.^13^ suggested that males keep them intact until they expose them to the degrading effects of sunlight during their spectacular courtship displays (see also Delhey *et al*.^19^), so that females could use the intensity (or, inversely, the degree of degradation) of the porphyrin-based salmon-pink coloration as a cue to determine if a male had copulated previously, as sperm quality decreases from the first to the last mating. More recently, a study on eagle owl (*Bubo bubo*) nestlings reported an association between the porphyrin content in feathers and body condition, suggesting that porphyrin coloration may signal their individual quality to parents^13^. For humans, porphyrins are only perceptible as pink fluorescence when stimulated with artificial UV light, and the extent and brightness of this fluorescence has been employed by researchers to determine the age of nightjars and owls^20,21^. Nonetheless, it has not been demonstrated that the species involved are actually able to see the pink fluorescence of porphyrins under natural levels of UV light, and their possible function(s) remain poorly understood due to the limited number of studies available.

**Figure 1:**
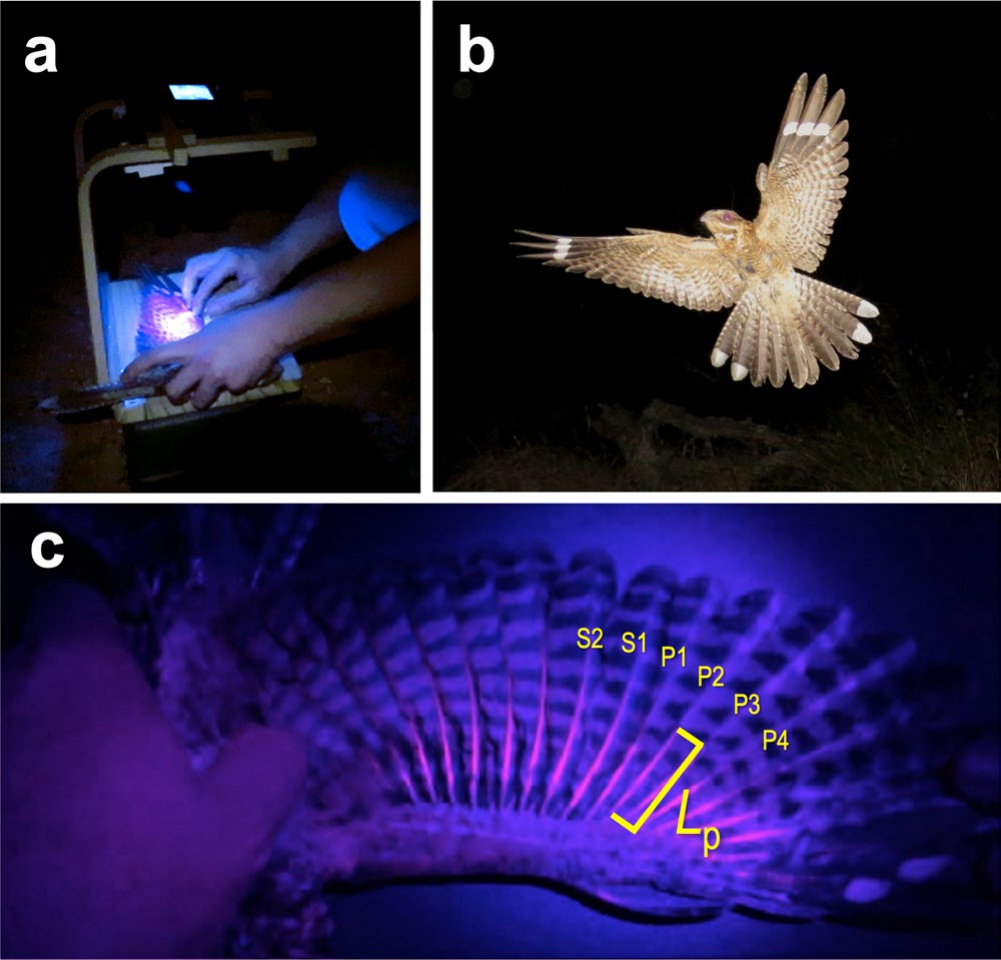
Photographs of red-necked nightjar plumage showing differences in perception of porphyrin-based coloration under natural and ultraviolet light. (**a**) Details of the wooden structure used to take standardized ventral photographs of nightjar plumage under a 395 nm light source. (**b**) Male red-necked nightjar during a courtship flight showing clearly visible white patches on wings and tail. Note that porphyrin-based coloration is not perceptible to the human eye under natural light. (**c**) Example of the under-side of a nightjar wing under ultraviolet-light irradiation, showing fluorescent sections along the rachis of exposed primaries and secondaries. The yellow line denotes the length of pink fluorescence (*L*_p_) for an individual feather (the second innermost primary, P2) and also serves to illustrate the differences in *L*_p_ among closely adjacent feathers (e.g. between S1 and P1). Photographs by Carlos Camacho.

Here, we investigated sources of variation in porphyrin-based pink fluorescence exhibited by nocturnal birds based on a well-studied population of red-necked nightjars in the Doñana Natural Park (Spain). Our aim was to assess the amount of pink fluorescence on the ventral side of nightjars according to (i) sex, (ii) age, (iii) body condition, (iv) time of the year (as a correlate of exposure to daylight in temperate latitudes), and (v) the extent of the only conspicuous part of the nightjar plumage (i.e. white wing patches), only visible when individuals open their wings^22^ (Fig. 1B). In addition, we discuss the possible role of porphyrins in the plumage of nightjars in relation to current functional hypotheses applied to any integumentary pigment deposited in either feathers or exposed skin^23^: (*i*) intraspecific communication, (*ii*) disguise –and not only camouflage, but also mimicry–, and (*iii*) physical protection, e.g. for thermoregulation or UV protection. Alternatively, porphyrins might just be a by-product of heme metabolism in the bird groups involved, with no adaptive value^17^.

## Results

We measured the amount of pink fluorescence for 69 individuals, including 30 first calendar-year birds (hereafter referred to as ‘juveniles’; 14 males, 16 females) and 39 adults (i.e. second calendar-year birds or older; 27 females and 12 males). Between 44% and 100% of the flight feathers examined in this study (N = 18) showed some extent of pink fluorescence, at a median of 14.5 feathers (Q1–Q3: 13–16.75) in juveniles, with a newly developed plumage, and 12 feathers (Q1–Q3: 10, 14.5) in adults.

**Figure 2:**
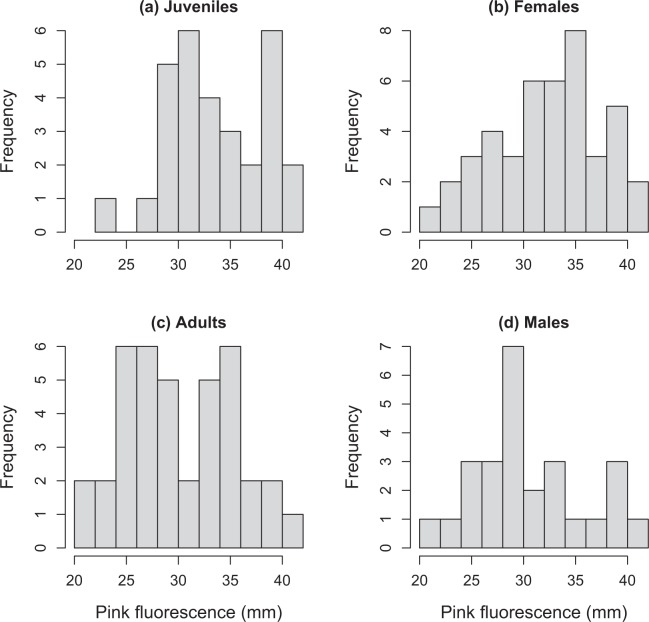
Frequency distribution of pink fluorescence in different segments of the study population of red-necked nightjars.

Juvenile nightjars displayed greater fluorescent patches than adults, as indicated by left-skewness in their frequency distribution relative to that of adults (K-S test, *D* = 0.344, *P* = 0.037; Fig. 2A,C). The total amount of pink fluorescence was only slightly greater in females than in males in both the juvenile (mean ± SE, Males: = 31.54 ± 2.04 mm; Females: 36.31 ± 2.51 mm) and the adult age-classes (Males: = 28.17 ± 1.91 mm; Females: 30.52 ± 1.12 mm), although a comparison of their frequency distributions showed marginally non-significant differences between sexes (age-classes pooled, *D* = 0.327, *P* = 0.062; Fig. 2B,D).

The amount of pink fluorescence did not correlate with the size of the white spots on the three outmost primaries in any age class (Table 1). Nevertheless, an analysis of the size of the white wing patches in the same sample of birds revealed that males had larger patches than females in both age classes (mean ± SE, Males: = 7.21 ± 0.24 mm^2^; Females: 4.68 ± 0.14 mm^2^. LM sex: *F*_1, 66_ = 97.44, *P* < 0.001, sex*age: *F*_1, 64_ = 0.4, *P* = 0.53).

**Table 1:** Results of the Linear Mixed Models analyzing the factors influencing the amount of pink fluorescence in juvenile and adult red-necked nightjars, controlling for the effects of the total number of individual feathers displaying pink fluorescence and the length of outermost primary.

	Estimate	S.E.	*d.f*.	*t*	*P*
**(a) Juvenile birds:** number of observations = 30, number of days = 10					
Intercept	34.214	1.10	30	31.14	< 0.001
Feathers with pink	5.542	1.13	30	4.90	** < 0.001**
Length P10	1.937	1.09	30	1.78	0.086
Body condition	2.488	1.13	30	2.20	**0.035**
Size of white wing patch	−2.179	1.49	26	−1.46	0.156
Week	2.398	1.16	30	2.15	**0.040**
Week^2	−0.307	0.97	30	−0.32	0.754
**(b) Adult birds:** number of observations = 39, number of days = 14					
Intercept	29.416	0.60	39	48.96	< 0.001
Feathers with pink	5.429	0.63	39	8.61	** < 0.001**
Length P10	−0.779	0.62	39	−1.25	0.219
Body condition	0.343	0.66	39	0.52	0.608
Size of white wing patch	−0.286	0.67	35	−0.43	0.670
Week	3.328	0.63	39	5.26	** < 0.001**
Week^2	1.372	0.69	39	2.00	0.053

Note that the sample size differs slightly for the models including the size of the wing patch because this trait was not measured for all individuals (see main text). Statistics and P-values of non-significant terms are those obtained by adding them individually to models containing only significant predictors and controlling variables regardless of their level of significance.

**Figure 3:**
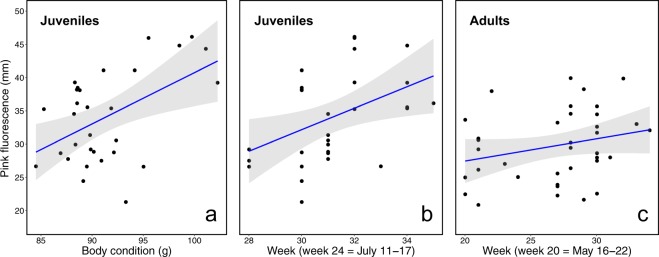
Effect of body condition and seasonality on the extent of pink fluorescence on the ventral surface of the wing of red-necked nightjars. **(a)** Body condition positively influenced the extent of pink fluorescence in juvenile (first calendar-year) nightjars. The extent of pink fluorescence increased throughout the breeding season in juvenile birds **(b)** and in adult birds. **(c)** Black circles are the fitted values from the LMMs, the blue solid line represents the regression line, and the shaded area indicates the 95% confidence interval.

For adults, there was no relationship between physical condition and the amount of pink fluorescence. However, this relationship was positive and statistically significant in juvenile birds (Table 1, Fig. 3a). On average, the amount of pink fluorescence in juveniles increased by ca. 20% for every 10-g increase in body mass (Fig. 3a). For both age classes we also found a significant increase in the amount of pink fluorescence as the breeding season progressed at an average rate of ca. 4% per week in juveniles and 1% per week in adults (Fig. 3b), although this increase tended to be non-linear in adults (Table 1).

## Discussion

The goal of this study was to investigate intrinsic and extrinsic sources of variation in the porphyrin-based fluorescence on the ventral surface of red-necked nightjars. Our main findings were: (*i*) there is considerable inter-individual variation in the amount of pink fluorescence of the plumage of juvenile and adult red-necked nightjars, (*ii*) greater differences in the amount of fluorescence exist between age classes than between sexes, (*iii*) the amount of fluorescence increases throughout the breeding season in both age classes, and (*iv*) juveniles, but not adults, in better condition display greater fluorescent patches on the under-side of their wings than those in poor condition, regardless of sex.

Nightjars and allies exhibit conspicuous sexual ornaments for visual signaling, such as white plumage patches and modified flight feathers^22,24,25^, although there is no confirmed evidence that these birds can perceive the pink fluorescence of porphyrins in response to the relatively weak levels of UV light that are naturally present during the night or at dusk and dawn. Nightjars and owls show similar patterns of signaling activity, and increase their vocal behavior under twilight and full-moon conditions^26–28^. During these periods, the UV portion of the light spectrum is enhanced relative to other wavelengths, and the efficiency of inconspicuous signals is thought to be boosted^16,26,27^. Eagle owls are assumed to have the capacity to perceive small increments in the red-pink reflectance of feathers due porphyrin-based fluorescence in dim light^13^. Based on the numerous convergences between owls and nightjars for both night living and day resting (e.g., cryptic plumages based on melanin patterns, modified barbules for silent flight, large eyes, emission of loud calls, use of white plumage patches for visual communication)^16,29^, then it is possible that nightjars can also perceive the fluorescence of porphyrins under natural light conditions, although further studies are required to confirm this possibility.

Recent evidence from different bird species, including parrots^30^, owls^13,20^, bustards^12^, and a few seabirds^31,32^ suggests that fluorescent colorations may be part of their visual communication toolbox. Galván *et al*.^13^ considered the signaling potential of plumage porphyrins in eagle owls, and found a positive correlation between the nutritional condition of nestlings and porphyrin concentration in feathers. Our results for juvenile nightjars, though not based on porphyrin concentration, but on the extent of the visible porphyrin patch, parallel those reported for the owlets^13^, and so we tentatively interpret them in the same manner: porphyrins in the plumage of juvenile nightjars might reveal their physical condition. We must acknowledge, however, that there is no proof that natural UV-light elicits enough fluorescence to be perceived by nightjars or other animals. Marshall & Jonhsen^17^ provided a checklist of five conditions for fluorescence to work as a visible signal. In the case of nightjars, three of those conditions are met, including that the fluorescent patches may be readily visible when individuals spread their wings, and that individuals perform specific behaviors on the ground that can help to display the fluorescent patches on the under-side of the wings. In addition, as explained above, nightjars are most active at sunset^28^, when the proportion of UV-light is expected to be higher. However, we do not know whether the excitation and emission wavelength ranges of the nightjar’s porphyrin are visually relevant, and we ignore the spectral sensitivity range of the species.

Most often, the UV-induced fluorescence of nestling plumage has been interpreted as a form of signaling genetic and phenotypic quality to parents^13,33^. Nonetheless, all juvenile nightjars measured in this study exceeded the age at which parents typically cease provisioning (35 days^34^), indicating that porphyrins, insofar as they can be perceived under natural light, might still act as a signal later in life. For instance, the expression of porphyrin-based coloration so early in life might aid in reproductive signaling in the year post-hatching. Nightjars are obligate migrants and start breeding in their first potential breeding season, even before the natal plumage is replaced^35,36^. First-year breeders should therefore retain any ornament and visual signal they might use to communicate their quality to females in their first potential breeding season. Nightjars would thus benefit from an early expression of sexual ornaments, including the white patches on remiges and rectrices^22^ and, possibly also, porphyrin patches on the ventral parts of their body.

Male and female nightjars analyzed in this study showed no dimorphism in the amount of pink fluorescence, a pattern also observed in flying squirrels^16^. However, the surface areas of the spots of the primaries did differ between the sexes, being larger in males than females. Achromatic plumage patches have proven important for nocturnal species in reproductive signaling, including the red-necked nightjar^22,24^ and, therefore, a correlation between the extent of white and pink fluorescence could be expected under the assumption that porphyrin-based coloration is a sexually selected signal. However, we found no such correlation. This result would appear to argue against the role of pink fluorescence in sexual communication, but it is still possible that these two traits signal different properties of the overall quality of an individual^37^.

Besides intraspecific communication, the pink fluorescence of nightjars could play a role in interspecific communication, possibly related to predator avoidance. For instance, pink fluorescence in New World flying squirrels has been proposed as a form of Batesian mimicry to escape predation from several co-occurring owl species that also display bright pink fluorescence on their ventral surfaces^16^. Nightjars rely on cryptic plumage patterns produced by melanin-based colors (brown and black) to avoid detection by diurnal predators, but their disruptive coloration poorly matches the uniformly-colored background of the roads that they use to forage, thus making nightjars vulnerable to nocturnal aerial (owls) and terrestrial (carnivores) predators^22^. Based on previous studies indicating that porphyrins and melanins may appear together in the plumage of nocturnal and crepuscular bird species^13,18^, it seems reasonable to think that the combination of pigments might provide a dual form of camouflage, involving conventional crypsis against diurnal predators through melanins, and an unusual case of porphyrin-mediated Batesian mimicry against owls, as suggested for fluorescent flying squirrels^16^.

The question remains as to why nightjars, and also owls, would resort to such an ephemeral and inconspicuous pigment, either as a form of communication or disguise. Galván *et al*.^13^ suggested that porphyrins offer nocturnal birds a subtle mechanism for intraspecific communication. For species that strive not to expose themselves to either prey or predators, the evolution of private communication channels can be expected (e.g. Stevens & Cuthill^38^). However, even the most cryptic species need to reveal themselves at some point in sexual/agonistic contexts. For this purpose, many bird species resort to restricted and facultative signaling strategies that provide a good balance between crypsis and conspicuousness^22^. These strategies include the white plumage patches and repetitive calls of nightjars and owls^25,29^ and, possibly also, the porphyrin-based coloration of the underwing surfaces. Because of their very location, the porphyrin patches of nightjars can be easily concealed when at rest and, perhaps, only owls and nightjars might be equipped to perceive them under poor light conditions^29^.

Our analyses also revealed that the extent of pink fluorescence peaked at the end of the breeding season in both juvenile and adult nightjars. Two different, non-functional, hypotheses may be advanced to explain this pattern. First, it is possible that porphyrin degradation is most pronounced at the beginning of the season, just around the summer solstice. Because of the protracted breeding period of nightjars (June-September^34^), the daily exposure time of chicks to sunlight decreases by 20% throughout the season, potentially resulting in greater levels of fluorescence in late-fledging chicks. Second, in the case of adults, increased exposure to sunlight might have contributed to the slight decrease in fluorescence expression observed before the summer solstice, but the onset of molt likely provides a more straightforward explanation for the increasing trend recorded from then onwards. Nightjars start molting during the chick-rearing period^34^ and, therefore, the proportion of newly grown feathers fully loaded with intact porphyrins increases gradually until molt is completed before migration departure. Last, the selective mortality of individuals showing smaller pink patches could also result in the apparent increase in fluorescence as the summer progresses, although the relatively high juvenile and adult survival rates recorded in this population argue against this possibility^36,39^.

More research into the biological significance of porphyrin-based colorations is sorely needed, but since the early work by Völker^11^, it is known that porphyrins are more prevalent in the plumage of nocturnal birds, with owls and caprimulgids accounting for about 5% of all avian species^40^. Due to their photolabile nature, the utility of porphyrins for communication could be partly lost in diurnal species, which typically resort to colorful combinations of bright pigments and structure to announce themselves to conspecifics. Thus, porphyrin-based colorations in diurnal birds, such as bustards and allies^12^ and the *Elanus* kites^18^, would be more the exception than the rule. Moreover, the proposed functions of porphyrins in those cases (e.g. cues during mate-choice and camouflage, respectively^12,18^), differ substantially from those suggested in nocturnal species, including signaling of individual status and predator avoidance through Batesian mimicry^13,16^.

Last, we believe that the next step to increase our knowledge of the origin and function of plumage porphyrins is to conduct research on the route followed by the porphyrins before deposition in feathers, and also to perform field experiments to corroborate or refute earlier findings. For instance, one could experimentally increase and/or decrease the extent of porphyrin patches in the nightjar or another nocturnal species and then quantify their mating success the following season. But, importantly, the amounts of pink fluorescence under natural levels of UV illumination need to be measured first, so that the ability of nightjars and other nocturnal species to perceive these potential signals under natural light conditions can be confirmed using behavioral experiments. Further research is also required to investigate additional functions of porphyrins outside a signaling context. For instance, porphyrins do not absorb significantly in the infrared, and could thus play a thermoregulatory role^41^. More studies on porphyrin-based coloration in birds and mammals would increase our understanding of the ecological significance of fluorescence and, more generally, of the evolution of visual communication.

## Material and Methods

### Study site and species

Fieldwork was conducted between 17 May and 16 September 2016 in an intensively (2008–2019) studied population of migratory red-necked nightjars in Doñana Natural Park, SW Spain (37°7′N, 6°33′W)^34,42^. Most nightjars arrive in the region in May, lay one or two clutches of 1–2 eggs between May and August, and head back to West Africa between September and October^34^. Males arrive from migration approximately one week before females, choose a territory, and vocalize to attract females. Male courtship is performed in front of the female and consists of a combination of visual (wing spreading) and acoustic (wing clapping) signals conducted in the males’ territories between dusk and dawn^43^. Nightjar chicks fledge at 18–22 days of age and, although they are able to forage for themselves, both parents regularly feed them for 2–3 weeks after they fledge^34^. Most juveniles return to their natal area to breed during their first potential breeding season, but around 20% of them may postpone reproduction until their second or even third year of life^36^.

Molt patterns are complex, but clear differences among age classes have been noted. Juvenile (first calendar-year) birds do not replace any of the primaries or rectrices at the natal sites, so all flight feathers are of the same generation. One remarkable aspect of the first plumage of nightjars is that sexual ornaments are expressed as soon as the outermost primaries and rectrices are visible^22,24^, possibly as a result of their tendency to breed before these feathers are replaced by adult ones^34^. Birds in their second calendar-year replace the central pair of rectrices, the outermost secondaries, and the innermost primaries during their first winter, as well as some additional primaries and rectrices after the first reproductive event^35^; therefore, two generations of feathers can be observed in second-year nightjars. During their second winter, nightjars replace the remaining feathers^35^ and undergo an extensive post-nuptial molt of up to 10–15 flight feathers before autumn migration^34^. From then on, the molt sequence is the same as described for second-year birds.

### Field procedures

We located and captured nightjars during nocturnal road-transect counts conducted once a week. Nightjars are sit-and-wait foragers and commonly use open spaces like roads as an observation platform to detect flying insects in the dark^44^. Even chicks, which are able to catch prey from an early age, move to roads located in the periphery of the nest-site^34^. Hence, periodic capture sessions along roads allowed us to capture a representative sample of the population. Capture sessions began 1 h after sunset and continued until the road transect (24 km) was sampled by driving a car at 30 km/h, which took between 3 and 6 hours depending on the number of captures. Every nightjar we encountered was dazzled by a spotlight and those that did not escape (approximately 50% success) were captured using a butterfly net^45^. To allow individual identification, we banded all nightjars with a numbered metal band. For each nightjar, we measured a set of morphological traits, including tail and wing length (±0.5 mm), length of outermost primary (±0.5 mm), keel length (±0.01 mm), and body mass (±0.1 g). We also determined their sex according to plumage characteristics^24^ and aged them as either first calendar-year (juvenile) or after first calendar-year (adult) following the criteria of Gargallo^35^. The primaries and rectrices of nightjars under 35 days old are still emerging from sheaths^34^, and so were excluded from the study to avoid underestimating the extent of porphyrin due to incomplete feather growth. Because of the high natal philopatry and recruitment rates in the study population, we knew the exact age of numerous birds marked as fledglings^36^.

Male and female nightjars share incubation duties, so brood patch development can be used as a reliable predictor of the breeding status in both sexes^46^. For males and females, we scored brood patches as active (females: carrying an egg or showing a completely bare patch with obvious blood vessels; males: completely bare patch) or inactive (males and females: forming feathered patch, regressing wrinkled patch, or completely absent). Molting stage was assessed as the number of rectrices and primary feathers at any molt stage (i.e. from new feathers just emerging to almost fully grown). Finally, we photographed all individuals in a standardized way (see below) and released them.

### Bird photographs and image analysis

To measure the amount of pink fluorescence and the size of white patches on primaries we used digital photography under standardized conditions and subsequent image analysis. C.C. and P.S-G. took all pictures used in this study. Note that, although there is no information on the type and relative concentration of porphyrins in nightjar feathers, the aim of this study was to understand the correlates and biological significance of porphyrin-based coloration and, therefore, we focused on functional aspects of the trait, rather than on its chemical composition. More specifically, we measured the amount of pink fluorescence along the rachis of primaries and secondaries.

For pink fluorescence, we photographed the under-side of the wings for two reasons. First, although porphyrins are prone to fast photodegradation^12^, exposure to sunlight of the underwing surfaces, and therefore, the degradation rate of porphyrins, should be limited in nocturnal species^20^. Second, it has been suggested that porphyrin-based coloration may be important in mate selection^12^. Measuring variation in the current amount of pink fluorescence on the under-side of the wings may be biologically relevant, as male nightjars spread the wings in front of prospective female mates during courtship flights from the ground (Fig. 1B).

For ventral photographs, taken under UV-light irradiation from a fixed distance of 25 cm, we placed each bird in exactly the same position relative to the camera and the UV light source. Two Morpilot UV flashlights (12-LED, 395 nm) mounted on a wooden structure were used as the UV light source (Fig. 1A), and the camera used was a Panasonic Lumix DMC-FZ200 f/2.8. Birds were placed on their back and held with the left wing outstretched by a second person, who at the same time bent the under wing-coverts down away from their natural lay to leave the rachis of primaries and secondaries exposed for the pictures (Fig. 1C). To standardize size, we also placed a mm-scaled stainless-steel ruler. Because the UV-light beams did not cover all primaries (N = 10) and secondaries (N = 13), the length of pink was measured for the 18 outermost remiges, including all 10 primaries and the 8 outermost secondaries. Our actual estimate of the amount of pink fluorescence was the summation of the lengths of pink rachis sections in the set of 10 primaries and 8 secondaries, to the nearest 0.5 mm (see refs. ^22^ and^24^ for a similar approach). All measurements of pink fluorescence were taken by the same person (JJN) using a stainless-steel ruler directly on the computer screen, after making sure that the size of the ruler used as a benchmark for image calibration matched that of the ruler placed on the screen.

For measurements of the white wing patches, we took a standardized photograph of the upper surface of one wing using the camera’s flash and the same mm-scaled stainless-steel ruler as a benchmark for calibration of the image. Each bird was held upside down onto a flat surface with either the left or right wing outstretched so that the patches on the outermost primaries (P10-P8 and, occasionally, also P7) were completely visible and measureable. Once in the lab, we measured the area (to the nearest 0.01 cm^2^) of the white patch on each of the three outermost primaries using ImageJ 1.50i software (Wayne Rasband National Institutes of Health, USA) and then summed the three measurements to obtain the total size of the trait. Measurements of the white patches were all taken by the same person (IR). Only the birds that were not molting the outermost primaries were measured for this trait (N = 71).

### Data analyses

To assess the factors influencing the extent of pink fluorescence on the under-side of the wings, we fitted Linear Mixed Models (LMM, normal error structure and identity link) using the package ‘lmerTest’^47^ of the program R version 3.5.1 (https://www.r-project.org). Conceptually, our response variable was the amount of pink fluorescence on the under-side of the wings – a trait that can be approximated as the surface area of a patch. However, this approach would result in overestimated values, because the amount of pink differs markedly even between adjacent feathers due to asynchronous molt of primaries and secondaries^20^. For this reason, we used as the response variable the sum of the lengths of pink across the rachis of primaries and secondaries (Fig. 2).

Because of the ephemeral nature of porphyrins, the expression of pink fluorescence is strongly conditional on the age of feathers themselves, such that only feathers of the most recent generations display fluorescence^12,20^. Nightjars are sensitive to the effects of stochastic environmental factors, such as drought or prey reduction, that determine the onset and extension of molt^34^, and this often results in irregular patterns of feather replacement^35^ that might influence the expression of pink fluorescence independently of intrinsic attributes of the individuals (e.g. body condition). Hence, to obtain an estimate of the amount of pink fluorescence relative to feathers of the same (most recent) generation and thus partly alleviate the potentially confounding effects of environmental stochasticity on the expression of this trait, we used the total number of individual feathers displaying pink fluorescence as a controlling variable. In addition, we included the length of outermost primary (P10) as a covariate in the models to control for the length of the feathers themselves.

Juvenile and adult nightjars display distinct molt strategies^35^ and also differ in important morphological traits, such as body mass, the length of tail and wing feathers, and the size of tail and wing spots^24,43^. Moreover, initial exploratory plots revealed significant age-related differences in the frequency distribution of the extent of fluorescence, as subsequently confirmed by a two-sample K-S test (see ‘Results’) using the function *ks.boot* (10,000 simulations) in the R-package ‘Matching’^48^. These differences appeared to be consistent across sexes, as revealed by an exploratory LMM based on all individuals (sex × age class: estimate ± SE = −4.25 ± 3.16, *P* = 0.178). Therefore, data on males and females were analyzed together, but we treated first calendar-year and adult nightjars separately in the models.

In all models, we included sex as a fixed effect and day of capture as a random (categorical) term to account for the potential effects of unmeasured environmental factors (e.g. cloudiness, ambient light levels) on the lighting of pictures. In addition, we fitted week number (week 1 = January 4–10 2016) and its quadratic term as continuous predictor variables to formally test for seasonal trends in the extent of pink fluorescence. Body mass corrected for structural size (keel length) and amount of food contained in the stomach, estimated through palpation of the abdomen as empty, ¼, ½, ¾ or full^46^, was included as a covariate (on the natural scale) to test for the effect of physical condition. In addition, to assess the possible role of porphyrin-based traits in mate choice decisions, we explored the association between the amount of pink fluorescence and the sum of the surfaces of the white spots on PP8–10, a sexual ornament that nightjars exhibit in their courtship displays^22,24^ (Fig. 1B). Nightjars exhibit strong sexual dimorphism in this trait^22,24^, and, therefore, sex and spot size could not be included at the same time in the models due to collinearity problems (Variance Inflation Factor, VIF > 4, calculated using the ‘car’ package^49^). Only size of the white wing patches was retained in the models, as sex-specific differences the amounts of pink fluorescence are analyzed through a comparison of frequency distributions using a two-sample K-S test as described above for juveniles and adults. Estimates of the level of collinearity among the other variables were all acceptable (mean VIF = 1.35, range: 1.12–1.77). Breeding status was not included in the model due to the relatively small number of adults showing an active brood patch (i.e. incubating individuals) at the time of capture (N = 10). Prior to running the models, we z-transformed all continuous predictors to a mean of zero and a standard deviation of one to achieve comparable estimates^50^.

Model simplification was carried out using both forward and backward selection based on likelihood ratio chi-square statistics, and both approaches produced the same results. To avoid misleading conclusions based on statistical artifacts, we systematically performed diagnostic statistics (e.g. inspection of residuals plotted against predicted values, examination of influential data points and assessment of interrelations among predictor variables). None of these showed obvious deviation from the assumptions of normality and homogeneity of residuals or revealed influential cases or outliers, confirming model stability (see above for a description of collinearity problems between sex and size of white wing patches).

### Experiments on live vertebrates

The authors declare that all procedures have been approved by the Andalusian Authority for Wildlife Protection, through the permit number: 2016107300002288/FQH/MDCG. This study did not involve threatened or endangered species and was carried out in accordance with national and international guidelines for care and use of animals.

## Data Availability

The data supporting the results of this study are available at https://digital.csic.es/handle/10261/193929.

## References

[1] Moore BA, Paul-Murphy JR, Tennyson AJ, Murphy CJ (2017). Blind free-living kiwi offer a unique window into the ecology and evolution of vertebrate vision. BMC Biol..

[2] Torres CR, Clarke JA (2018). Nocturnal giants: evolution of the sensory ecology in elephant birds and other palaeognaths inferred from digital brain reconstructions. Proc. R. Soc. London B Biol. Sci..

[3] Ödeen A, Håstad O, Alström P (2011). Evolution of ultraviolet vision in the largest avian radiation - the passerines. BMC Evol. Biol..

[4] Prum, R. O. Anatomy, physics, and evolution of avian structural colors, In *Bird Coloration*, Vol. I. *Mechanisms and Measurements* (eds. Hill, G. E. & McGraw, K.) 295–353 (Harvard University Press, Cambridge MS 2006).

[5] Negro JJ, Margalida A, Hiraldo F, Heredia R (1999). The function of the cosmetic coloration of bearded cultures: when art imitates life. Anim. Behav..

[6] Negro JJ, Margalida A, Torres MJ, Grande JM, Hiraldo F, Heredia R (2002). Iron oxides in the plumage of bearded vultures. Medicine or cosmetics? Anim. Behav..

[7] McGraw, K. J. Mechanics of uncommon colors: pterins, porphyrins and psittacofulvins, In *Bird Coloration*, Vol. I. *Mechanisms and Measurements* (eds. Hill, G. E. & McGraw, K.) 354–398 (Harvard University Press, Cambridge MS (2006).

[8] Negro JJ, Sarasola JH, Fariñas F, Zorrila I (2006). Function and occurrence of facial flushing in birds. Comp. Biochem. Physiol. A..

[9] Goldberg A, Ashenbrucker H, Cartwright GE, Wintrobe MM (1956). Studies on the biosynthesis of heme *in vitro* by avian erythrocytes. Blood..

[10] Derrien E, Turchini J (1925). Nouvelles observations des fluorescences rouges chez les animaux. CR. Séances Soc. Biol..

[11] Völker O (1938). Porphyrin in vogelfedern. J. Ornithol..

[12] Galván I, Camarero PR, Mateo R, Negro JJ (2016). Porphyrins produce uniquely ephemeral animal colouration: a possible signal of virginity. Sci. Rep..

[13] Galván I, Delgado MM, Camarero PR, Mateo R, Lourenço R, Penteriani V (2018). Feather content of porphyrins in Eurasian eagle owl (*Bubo bubo*) fledglings depends on body condition and breeding site quality. Integr. Zool..

[14] Stachel SJ, Stockwell SA, Van Vranken DL (1999). The fluorescence of scorpions and cataractogenesis. Chem. Biol..

[15] Goutte S (2019). Intense bone fluorescence reveals hidden patterns in pumpkin toadlets. Sci. Rep..

[16] Kohler AM, Olson ER, Martin JG, Anich PS (2019). Ultraviolet fluorescence discovered in New World flying squirrels (*Glaucomys*). J. Mammal..

[17] Marshall Justin, Johnsen Sonke (2017). Fluorescence as a means of colour signal enhancement. Philosophical Transactions of the Royal Society B: Biological Sciences.

[18] Negro JJ, Bortolotti GR, Mateo R, García IM (2009). Porphyrins and pheomelanins contribute to the reddish juvenal plumage of black-shouldered kites. Comp. Biochem. Phys. B..

[19] Delhey K, Peters A, Kempenaers B (2007). Cosmetic coloration in birds: occurrence, function and evolution. Am. Nat..

[20] Weidensaul CS, Colvin BA, Brinker DF, Huy JS (2011). Use of ultraviolet light as an aid in age classification of owls. Wilson J. Ornithol..

[21] Blythman MD, Sansom JL (2016). Use of ultraviolet light to help age nightjars, owlet-nightjars, frogmouths and owls. Corella..

[22] Aragonés J, De Reyna LA, Recuerda P (1999). Visual communication and sexual selection in a nocturnal bird species, *Caprimulgus ruficollis*, a balance between Crypsis and conspicuousness. Wilson J. Ornithol..

[23] Negro JJ, Finlayson C, Galván I (2018). Melanins in Fossil Animals: Is It Possible to Infer Life History Traits from the Coloration of Extinct Species?. Int. J. Mol. Sci..

[24] Forero MG, Tella JL, García L (1995). Age-related evolution of sexual dimorphism in the Red-necked Nightjar *Caprimulgus ruficollis*. J. Ornithol..

[25] Cleere, N. Nightjars: A Guide to Nightjars and related birds. A&C Black (2010).

[26] Penteriani V, Delgado MM (2009). The dusk chorus from an owl perspective: eagle owls vocalize when their white throat badge contrasts most. PLoS ONE.

[27] Penteriani V, Delgado MM, Campioni L, Lourenco R (2010). Moonlight makes owls more chatty. PLoS ONE.

[28] Reino L, Porto M, Santana J, Osiejuk TS (2015). Influence of moonlight on nightjars’ vocal activity: a guideline for nightjar surveys in Europe. Biologia.

[29] Penteriani V, Delgado MM, Alonso-Alvarez C, Sergio F (2006). The importance of visual cues for nocturnal species: eagle owls signal by badge brightness. Behav. Ecol..

[30] Arnold KE, Owens IP, Marshall NJ (2002). Fluorescent signaling in parrots. Science..

[31] Dunning J (2018). Photoluminescence in the bill of the Atlantic Puffin *Fratercula arctica*. Bird Study..

[32] Wilkinson, B. P., Johns, M. E. & Warzybok, P. Fluorescent ornamentation in the Rhinoceros Auklet *Cerorhinca monocerata. Ibis*, 10.1111/ibi.127155 (2019).

[33] Galván I, Amo L, Sanz JJ (2008). Ultraviolet‐blue reflectance of some nestling plumage patches mediates parental favouritism in great tits *Parus major*. J. Avian Biol..

[34] Camacho C (2013). Tropical phenology in temperate regions: extended breeding season in a long-distance migrant. The Condor..

[35] Gargallo G (1994). Flight feather moult in the Red-necked Nightjar *Caprimulgus ruficollis*. J. Avian Biol..

[36] Camacho C (2014). Early age at first breeding and high natal philopatry in the Red‐necked Nightjar *Caprimulgus ruficollis*. Ibis..

[37] Møller AP, Pomiankowski A (1993). Why have animals got multiple sexual ornaments?. Behav. Ecol. Sociobiol..

[38] Stevens M, Cuthill IC (2007). Hidden messages: are ultraviolet signals a special channel in avian communication?. BioScience..

[39] Forero MG, Tella JL, Oro D (2001). Annual survival rates of adult Red-necked Nightjars *Caprimulgus ruficollis*. Ibis..

[40] Handbook of the Birds of the World and BirdLife International. Handbook of the Birds of the World and BirdLife International Digital Checklist of the Birds of the World. Version 9.1, http://datazone.birdlife.org/species/taxonomy (2017).

[41] Bakken GS, Vanderbilt VC, Buttemer WA, Dawson WR (1978). Avian eggs: thermoregulatory value of very high near-infrared reflectance. Science..

[42] De Felipe M, Sáez-Gómez P, Camacho C (2019). Environmental factors influencing road use in a nocturnal insectivorous bird. Eur. J. Wildl. Res..

[43] Sáez-Gómez, P. & Camacho, C. Chotacabras Cuellirrojo, *Caprimulgus ruficollis*, In *Enciclopedia Virtual de los* Vertebrados Españoles. (eds. Salvador, A. & Morales, M.). Museo Nacional de Ciencias Naturales, Madrid, http://www.vertebradosibericos.org (2016).

[44] Jackson HD (2003). A field survey to investigate why nightjars frequent roads at night. Ostrich..

[45] Jackson HD (1984). Finding and trapping Nightjars. Bokmakierie..

[46] Camacho C, Palacios S, Sáez P, Sánchez S, Potti J (2014). Human-induced changes in landscape configuration influence individual movement routines: lessons from a versatile, highly mobile species. PLoS One..

[47] Kuznetsova, A., Brockhoff, P. B. & Christensen, R. H. B. lmerTest: tests in linear mixed effects models. R package, https://cran.r-project.org/web/packages/lmerTest/index.html (2016).

[48] Sekhon, J. S. Multivariate and propensity score matching software with automated balance optimization: the matching package for R. Journal of Statistical Software 42, https://sekhon.berkeley.edu/papers/MatchingJSS.pdf (2011).

[49] Fox J, Friendly M, Weisberg S (2013). Hypothesis tests for multivariate linear models using the car package. R. J..

[50] Schielzeth H (2010). Simple means to improve the interpretability of regression coefficients. Methods Ecol. Evol..

